# Novel Plasma Biomarkers Associated with Future Peripheral Atherosclerotic Disease and Abdominal Aortic Aneurysm—Insights from Contemporary Prospective Studies from the Malmö Diet and Cancer Study

**DOI:** 10.3390/biom14070844

**Published:** 2024-07-13

**Authors:** Stefan Acosta, Shahab Fatemi, Moncef Zarrouk, Anders Gottsäter

**Affiliations:** 1 Department of Clinical Sciences, Lund University, 20213 Malmö, Sweden; shahab.fatemi@med.lu.se (S.F.); moncef.zarrouk@med.lu.se (M.Z.); anders.gottsater@med.lu.se (A.G.); 2 Vascular Centre, Department of Cardiothoracic and Vascular Surgery, Skåne University Hospital, Ruth Lundskogsgata 10, 20502 Malmö, Sweden; 3 Department of Internal Medicine and Emergency Medicine, Skåne University Hospital, 20502 Malmö, Sweden

**Keywords:** plasma biomarkers, incident cardiovascular disease, lower extremity arterial disease, carotid artery stenosis, abdominal aortic aneurysm, prospective study, Malmö Diet and Cancer Study

## Abstract

Introduction: The potential utility of inflammatory and hemodynamic plasma biomarkers for the prediction of incident lower extremity arterial disease (LEAD), carotid artery stenosis (CAS), isolated atherosclerotic disease without concomitant abdominal aortic aneurysm (AAA), and isolated AAA without concomitant atherosclerotic disease has not yet been integrated in clinical practice. The main objective of this prospective study was to find predictive plasma biomarkers for cardiovascular disease and to evaluate differences in plasma biomarker profiles between asymptomatic and symptomatic CAS, as well as between isolated atherosclerotic disease and isolated AAA. Methods: Blood samples collected at baseline from participants in the prospective Malmö Diet and Cancer study (MDCS) cardiovascular cohort (n = 5550 middle-aged individuals; baseline 1991–1994) were used for plasma biomarker analysis. Validation of each incident cardiovascular diagnosis was performed by random sampling. Cox regression analysis was used to calculate hazard ratios (HRs) per one standard deviation increment of each respective log-transformed plasma biomarker with 95% confidence intervals (CI). Results: Adjusted lipoprotein-associated phospholipase A2 (Lp-PLA_2_) activity (HR 1.33; CI 1.17–1.52) and mass (HR 1.20; CI 1.05–1.37), C-reactive protein (CRP) (HR 1.55; CI 1.36–1.76), copeptin (HR 1.46; CI 1.19–1.80), N-terminal pro-B-type natriuretic peptide (N-BNP) (HR 1.28; 1.11–1.48), and cystatin C (HR 1.19; 95% 1.10–1.29) were associated with incident symptomatic LEAD. Adjusted N-BNP (HR 1.59; CI 1.20–2.11), mid-regional proadrenomedullin (HR 1.40; CI 1.13–1.73), cystatin C (HR 1.21; CI 1.02–1.43), and CRP (HR 1.53; CI 1.13–1.73) were associated with incident symptomatic but not asymptomatic CAS. Adjusted HR was higher for Lp-PLA_2_ (mass) for incident isolated AAA compared to for isolated atherosclerotic disease. Conclusions: Plasma biomarker profile data support that subclinical vascular inflammation and cardiovascular stress seem to be relevant for the development of atherosclerotic disease and AAA.

## 1. Introduction

### 1.1. Biomarkers

Biomarkers can be used to determine whether a patient has a particular medical condition for which treatment may be indicated or if an individual free of a particular disease will develop that disease in the future. In clinical practice, no biomarker test has perfect clinical and analytical performance with 100% sensitivity and 100% specificity.

To be of clinical value in relation to cardiovascular diseases such as lower extremity arterial disease (LEAD), carotid artery disease (CAS), and abdominal aortic aneurysm (AAA), a biomarker must be stable, reliably measurable with reasonable cost and effort and give additional relevant information on diagnosis or prognosis when combined with the diagnostic methods already used in clinical practice. It is often beneficial to combine the assessment of several different biomarkers due to large intra-individual variations [1].

### 1.2. Old and New Plasma Biomarkers for Cardiovascular Disease

This review covers both older plasma biomarkers, such as C-reactive protein (CRP), and more novel plasma biomarkers, such as lipoprotein-associated phospholipase (Lp-PLA_2_) (mass and activity), proneurotensin, mid-regional proatrial natriuretic peptide (MR-proANP), mid-regional proadrenomedullin (MR-proADM), N-terminal pro-B-type natriuretic peptide (NT-proBNP), copeptin, and cystatin C. An overview of cell origin, type of marker, and disease marker of these plasma biomarkers is shown in Table 1 [2,3,4,5,6,7,8,9,10,11,12,13,14,15,16,17,18,19,20,21,22,23,24,25,26,27].

### 1.3. The Malmö Diet and Cancer Study Cohort—Cardiovascular Arm

A total of 71,104 middle-aged men and women from Malmö born between 1923 and 1950 (age range 45 to 73 years) were invited to participate in the population-based Malmö Diet and Cancer Study (MDCS) [28], but only 30,046 accepted and underwent all examination procedures [29]. Among the MDCS participants, a random sample (14,000 individuals) was invited to undergo additional evaluations, including blood sampling for future studies, but only 6103 participants were included in this MDCS–cardiovascular arm (MDCS-CV) cohort between 1991 and 1994 [28].

### 1.4. Objectives

The potential utility of inflammatory and hemodynamic plasma biomarkers for prediction of incident LEAD, CAS, isolated atherosclerotic disease without concomitant AAA, and isolated AAA without concomitant atherosclerotic disease has not yet been integrated into clinical practice. The main objective of this prospective study was to find predictive plasma biomarkers for cardiovascular disease and to evaluate differences in plasma biomarker profiles between asymptomatic and symptomatic CAS, as well as between isolated atherosclerotic disease (AD) and isolated AAA.

## 2. Methods

### 2.1. Study Design

This is a summary of five contemporary published prospective cohort studies on plasma biomarkers and incident cardiovascular disease by using data from the MDCS-CV [30,31,32,33,34,35].

### 2.2. Plasma Biomarkers

Plasma biomarkers were measured from fasting plasma samples frozen at −80 °C immediately after collection [36]. An overview of laboratory measurements, assays, manufacturers, and coefficients of variation (CV) are shown in Table 2 [22,36,37,38,39].

### 2.3. Endpoint Ascertainment

All subjects free of respective cardiovascular disease were followed up from the baseline examination until the first respective cardiovascular event (LEAD, CAS, coronary artery disease, atherothrombotic ischemic stroke, or AAA), mortality, emigration from Sweden, or end of follow-up on 31 December 2016. Individuals from the MDCS-CV with a first registered diagnosis of respective cardiovascular disease were identified from Swedish registers (the Inpatient and Outpatient Registries and the Cause of Death Register) by linkage of the unique 10-digit personal identification number. Diagnoses are coded using a Swedish revision of the International Classification of Diseases (ICD), versions 8, 9, and 10. Isolated AD was defined as AD without concomitant AAA, and isolated AAA as AAA without concomitant AD.

### 2.4. Validation of Cardiovascular Disease

One hundred randomly sampled patients with incident LEAD, CAS, and AAA identified in MDCS-CV were validated. Ninety-seven percent had symptomatic LEAD, advanced LEAD since 69 had chronic limb-threatening ischemia, and 13 had acute lower limb ischemia. Of patients with CAS, 99 had CAS, of which 57 had symptomatic CAS and 42 had asymptomatic CAS. Ninety-seven percent (96/99) had acute myocardial infarction. The diagnosis of ischemic stroke was confirmed in 89% (87/98). Among the 87 with ischemic stroke, 31 (35.6%) had embolization due to atrial fibrillation (AF). Patients registered with AF prior to or simultaneously (±30 days) to ischemic stroke were labeled as AF-related ischemic stroke and excluded. AF-related ischemic strokes were followed up until the date of incident atrial fibrillation. In this way, non-embolic atherothrombotic stroke was selected as the endpoint. Ninety-five percent had AAA, four had aneurysm formation outside of the abdominal aorta or pseudoaneurysm in the abdominal aorta, and only one patient had no aneurysmatic disease.

### 2.5. Statistics

Plasma biomarkers and confounders for incident cardiovascular disease were assessed using multi-variable Cox regression models, and hazard ratios (HRs) were expressed per one standard deviation (SD) increment of each respective log-transformed plasma biomarker (skewed distributed) in the Cox regression models. No imputation of data was made when there was missing data. Cumulative incidences of cardiovascular disease were analyzed using the Kaplan–Meier method. The log-rank test was used in the comparison between sex. Analyses were performed using SPSS for Windows, version 26.0 (SPSS Inc., Chicago, IL, USA); *p*-values less than 0.05 were considered significant.

### 2.6. Ethics

The scientific work reported was performed after appropriate approval of the following ethical applications: Dnr LU 51-90, approved 14 February 1990, Dnr 166-2007, approved 12 April 2007, Dnr 2009/633, approved 19 November 2009, Dnr 2013/566, approved 27 August 2013.

## 3. Results

### 3.1. Cumulative Incidence of Incident Cardiovascular Diseases

**Figure 1:**
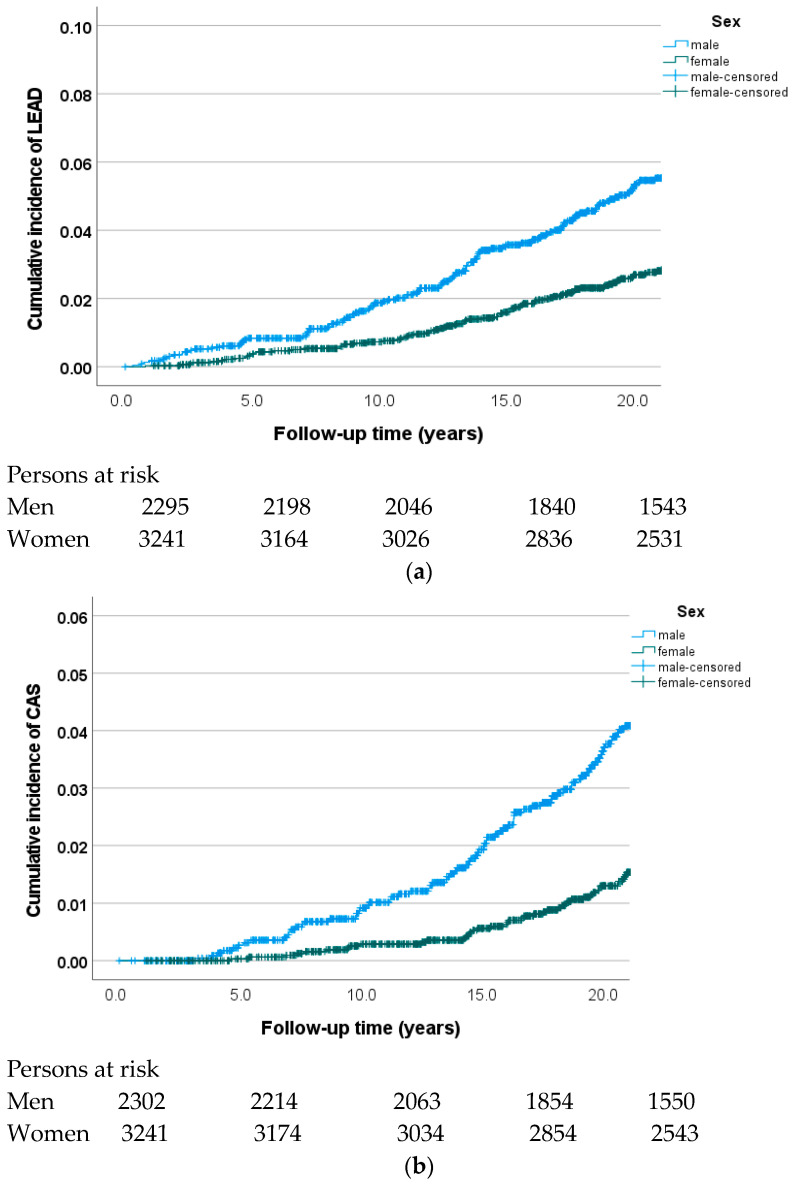

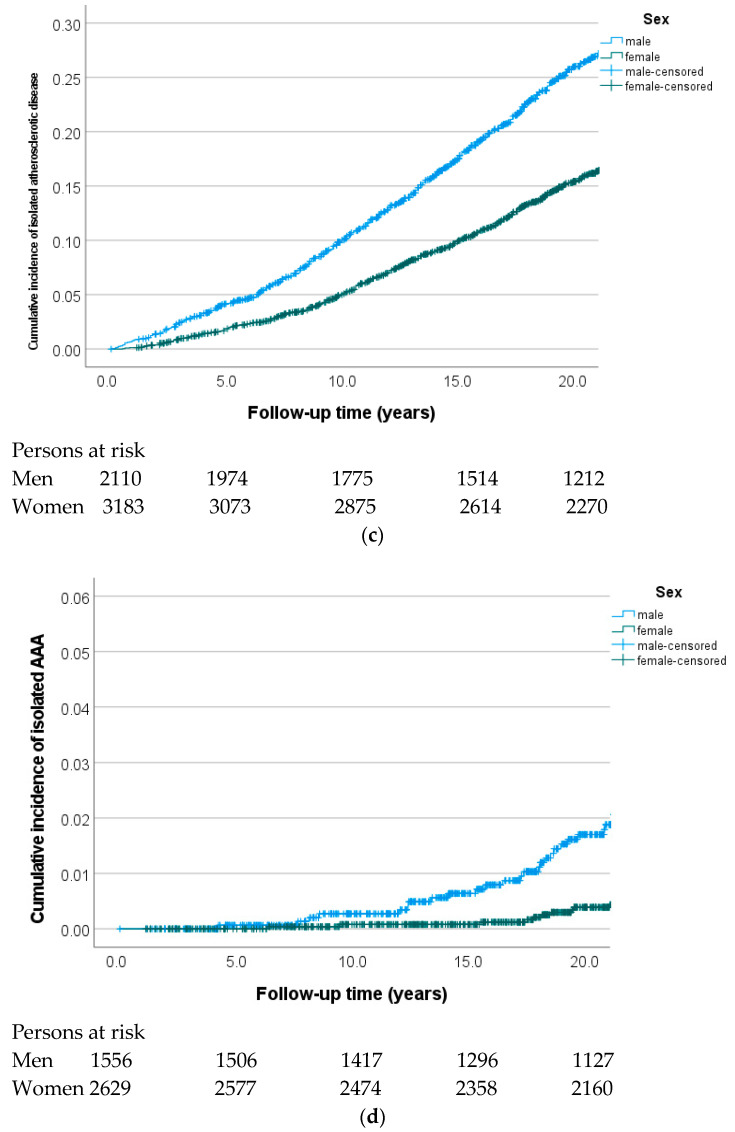
Cumulative incidence of symptomatic LEAD (**a**), CAS (**b**), AD (**c**), and AAA (**d**) in relation to sex among participants in the Malmö Diet and Cancer cohort.

The cumulative incidence of LEAD during a median follow-up of 23.4 years (interquartile range [IQR] 19.4–24.3) was 4.4% (244/5550); 5.9% (137/2307) for men and 3.3% (107/3243) for women (*p* < 0.001, Figure 1a). The cumulative incidence of CAS was 2.3% (125/5543), 3.4% (75/2227) in men, and 1.5% (50/3316) in women (*p* < 0.001) during a median follow-up of 23.4 years (IQR 19.5–24.3) (Figure 1b). During a median follow-up of 23.1 years (IQR 16.3–24.2), the cumulative incidence of isolated AD without concomitant AAA was 22.2% (1196/5381); 28.6% (622/2178) in men and 17.9% (574/3203) in women (*p* < 0.001) (Figure 1c). The cumulative incidence of isolated AAA without concomitant AD was 1.6% (88/5381), and 68 (77.3%) of study subjects diagnosed with AAA were men (*p* < 0.001 compared to AAA in women) (Figure 1d).

### 3.2. Plasma Biomarkers Associated with Incident Cardiovascular Disease

In multi-variable Cox regression analysis adjusted for conventional risk factors, Lp-PLA_2_ activity, Lp-PLA_2_ mass, CRP, Copeptin, N-BNP, and cystatin C were all independently associated with incident LEAD during follow-up (Table 3). NT pro-BNP, MR-proADM, cystatin C, and CRP at baseline were independently associated with incident symptomatic CAS during follow-up, whereas no associations were found between plasma biomarkers and incident asymptomatic CAS (Table 4). Lp-PLA_2_ (activity) and MR-proADM were both associated with incident-isolated AD and incident-isolated AAA during follow-up. NT pro-BNP, copeptin, cystatin C, proneurotensin, and CRP were associated only with incident-isolated AD, whereas Lp-PLA_2_ (mass) was associated only with incident-isolated AAA (Table 5). Adjusted HR for Lp-PLA_2_ (mass) (HR 1.53, 95% CI 1.14–2.04 vs. HR 1.05, 95% CI 0.99–1.12) was higher for incident isolated AAA compared to incident isolated AD, respectively.

## 4. Discussion

Elevated plasma levels of both inflammatory biomarkers such as Lp-PLA_2_ activity and mass, CRP, and copeptin, and cardiac and renal markers such as NT pro-BNP and cystatin C could all be considered markers of subclinical disease long time before the diagnosis of LEAD advanced enough to cause symptoms and hospitalization. A combination of multiple biomarkers [1] was also found to be useful, and a score taking high levels of copeptin, NT pro-BNP, and cystatin C into account could be used to predict LEAD [31].

It is important to note that 82% of patients in whom the diagnosis of LEAD was validated had either acute or chronic limb-threatening ischemia, and no conclusions can, therefore, be drawn from the present results regarding the prediction of less severe forms of LEAD not necessitating hospitalization. However, inflammatory markers have been previously related to intermittent claudication [40,41]. We cannot draw any conclusions from our findings regarding the potential importance of elevated biomarker levels for limb prognosis or mortality in patients in whom LEAD has already been diagnosed. Such relationships are known to exist, at least for CRP [42] and NT-pro BNP [43].

NT-pro BNP, MR-proADM, cystatin C, and CRP were all independently associated with the occurrence of symptomatic CAS, in addition to their previously documented associations with coronary artery disease and congestive heart failure [36]. As stroke incidence in CAS patients is low [44,45,46], selection of proper candidates for carotid artery surgery among patients in whom a CAS has so far remained asymptomatic is important. As subjects developing symptomatic CAS underwent sampling several years before the detection of CAS, the utility of the markers to predict neurological symptoms within the near future needs to be further investigated. The utility of a combination of the above four markers together with ultrasound plaque features [47] to predict neurological symptoms needs to be prospectively tested in a cohort undergoing modern pharmacological treatment [48] for an established asymptomatic CAS.

There seem to be complex and significant differences between the pathophysiology in symptomatic CAS featuring active atherosclerotic embolization and cerebral events and asymptomatic CAS with dormant atherosclerotic plaques [49,50]. The different plasma biomarker profiles might also indicate that individuals with incident symptomatic CAS have more generalized and potentially progressive subclinical atherosclerosis already at baseline compared to those developing incident asymptomatic CAS.

Plasma biomarker profiles for incident-isolated AD and incident-isolated AAA had several important differences despite their many similarities. Among the biomarkers, both the inflammatory marker Lp-PLA_2_ activity and the vasoactive marker MR-proADM were associated with both incident-isolated AD and incident-isolated AAA and could, therefore, be regarded as general indicators of increased risk for future vascular disease. The fact that Lp-PLA_2_ mass was more elevated in those developing isolated AAA than in those developing isolated AD might be interpreted as the development of AAA having a more distinct component of vascular inflammation. Both Lp-PLA_2_ activity and mass [51] and MR-proADM [52] have previously been established as biomarkers of future AAA hospitalization in MDCS subjects, but in these previous reports, AAA incidence was not evaluated as isolated AAA without concomitant AD. Interestingly, however, there might apparently be differences in biomarker patterns regarding the prediction of either small AAA or large AAA requiring hospitalization or surgery. The same markers predicted neither aortic dilatation nor asymptomatic aneurysm when male MDCS participants underwent ultrasonic screening for AAA at age 65 [53], suggesting that they might either not be relevant until closer to AAA diagnosis or only for prediction of larger AAA. On the other hand, the present review results do not allow us to draw any conclusion regarding the proposed relationships [54,55] between biomarkers and AAA growth.

NT pro-BNP, copeptin, cystatin C, proneurotensin, and CRP, on the other hand, predicted only the outcome variable incident isolated atherosclerotic disease, i.e., a wider set of cardiovascular manifestations. Apart from NT pro-BNP [56], none of the other plasma biomarkers were found to be associated with future AAA in patients with concomitant AAA and AD. Taken together, the differences in plasma biomarker profile long before diagnosis of either AD or AAA might be interpreted that these cardiovascular diseases are different disease entities with at least partly different pathophysiologies.

There are several limitations of the papers that contribute to this study. Firstly, only a few of the many biomarkers with potential importance for the prediction of LEAD, CAS, atherosclerotic disease, and AAA have been assessed. While the evaluated protein biomarkers functionally mainly can be categorized as belonging to the inflammation and hemodynamic system, there were no biomarkers that had a pronounced primary coagulation [57] profile. The storage of samples before analysis and the lack of meticulous baseline assessment of LEAD, CAS, and AAA are other limitations. Exclusion of subjects with prevalent disease already at baseline was performed by registry data and patient files, which is a rough method mainly identifying a few patients with symptomatic cardiovascular disease. Re-invitation of study participant survivors for objective detection and verification of the development of vascular manifestations would also have been valuable, as this would have captured both asymptomatic atherosclerosis and changes in risk factor status and medication. During the last decade, the declining prevalence of smoking and the improved pharmacological treatment among individuals with cardiovascular disease might well have impacted the cumulative incidence of AAA and the extent of atherosclerotic disease [58]. The present study design only allows us to report incident diseases of enough clinical importance to cause an episode of hospitalization to be registered in national registries.

Major strengths of the present report, on the other hand, were the longitudinal study design, the inclusion of healthy middle-aged individuals, and the long follow-up of 23.4 years. Additionally, thorough validation of patient files from 100 patients in each of the different disease groups confirmed that hospital diagnoses were accurate in the vast majority of cases. It must also be emphasized that the associations between different biomarkers and manifestations of vascular disease were independent of conventional, well-established risk factors, such as smoking, blood pressure and lipid levels, and glycemic status. On the other hand, we did not adjust for other risk markers of potential importance, such as nutritional factors [59], psychosocial stress [60], and family history [61]. First-degree relatives of MDCS subjects have been further examined [62]; however, the extraction of registry data regarding hospitalizations for AD in this group has recently been ethically approved, potentially enabling such analysis in the future.

Investigation of circulating biomarkers related to incident AD or aneurysm might potentially become a feasible screening alternative in primary care to select individuals for more thorough investigation and preventive lifestyle and/or pharmacological measures to counteract cardiovascular risk factors at an early stage before the development of manifest disease.

In conclusion, CRP and Lp-PLA2 activity and mass, NT pro-BNP, cystatin C, and copeptin are independently associated with incident LEAD during long-term follow-up. CRP, NT pro-BNP, cystatin C, and MR-proADM are independently associated with incident symptomatic CAS, whereas no plasma biomarker was associated with incident asymptomatic CAS. Different plasma biomarker patterns predict incident-isolated AAA and incident-isolated AD during long-term follow-up.

## Figures and Tables

**Table 1:** Overview of plasma biomarkers.

Name	Cell Origin	Type of Marker	Disease Marker
CRP [2,3,4,5]	Liver	Inflammatory	Cardiovascular Obesity Diabetes
Lp-PLA_2_ [6,7,8,9,10,11] (mass)	Monocytes	Inflammatory	Atherogenesis
Lp-PLA_2_ (activity)	Monocytes	Inflammatory	Atherogenesis
Proneurotensin [12]	Gut	Fat metabolism	Satiety Obesity regulation
MR-proADM [13,14,15]	Vascular smooth muscle and endothelial cells, cardiomyocytes	Vasodilatation	Endothelial dysfunction
MR-proANP [13,14,15]	Atria	Cardiac	Heart failure, volume overload
NT pro-BNP [16,17,18,19]	Atria	Cardiac	Heart failure, volume overload
Copeptin [20,21,22]	Hypothalamus	Inflammatory	Heart failure, acute myocardial infarction, ischemic stroke
Cystatin C [23,24,25,26,27]	Body fluids	Renal	Kidney failure

CRP: C-reactive protein, Lp-PLA_2_ (activity and mass): lipoprotein-associated phospholipase A2, MR-proADM: mid-regional proadrenomedullin, MR-proANP: mid-regional proatrial natriuretic peptide, N pro-BNP: N-terminal pro-B-type natriuretic peptide.

**Table 2:** Overview of laboratory measurements.

Name	Assay	Manufacturer	CV (%)
CRP	High sensitivity Tina-quant^®^ latex	Roche Diagnostics, Rotkreuz, Switzerland	4.59
Lp-PLA_2_ (mass) [37]	Sandwich enzyme immuno-	diaDexus Inc., San Francisco, California, United States	4.62
Lp-PLA_2_ (activity) [37]	Enzyme-linked immunosorbent	Non-commercial	5.78
Proneurotensin [38]	Chemiluminescence	Non-commercial	4.1–6.2
MR-proADM [39]	Immunoluminometric sandwich	Brahms AG, Hennigsdorf, Germany	≤10
MR-proANP	Immunoluminometric sandwich	Brahms AG, Hennigsdorf, Germany	≤10
NT pro-BNP [36]	Automated Dimension Vista Intelligent Lab System	Siemens diagnostics, Erlangen, Germany	2.7
Copeptin [22]	Chemiluminescence	Brahms AG, Hennigsdorf, Germany	<20
Cystatin C	Particle-enhanced immune-nephelometric	Siemens diagnostics, Erlangen, Germany	4.3

CRP: C-reactive protein, Lp-PLA_2_ (activity and mass): lipoprotein-associated phospholipase A2, MR-proADM: mid-regional proadrenomedullin, MR-proANP: mid-regional proatrial natriuretic peptide, NT pro-BNP: N-terminal pro-B-type natriuretic peptide, CV: coefficient of variation.

**Table 3:** Hazard ratios for plasma biomarkers associated with incident LEAD.

Plasma Inflammatory Biomarkers	PAD n = 244. HR * (95% CI)
C-reactive protein (n = 5300)	1.55 (1.36–1.76)
Proneurotensin (n = 4627)	0.94 (0.80–1.09)
Lipoprotein-associated phospholipase A_2_ (mass) (n = 5390)	1.20 (1.05–1.37)
Lipoprotein-associated phospholipase A_2_ (activity) (n = 5395)	1.33 (1.17–1.52)
Plasma hemodynamic biomarkers	
Cystatin C (n = 5150)	1.19 (1.10–1.29)
Copeptin (n = 5248)	1.46 (1.19–1.80)
N-terminal pro-B-type natriuretic peptide (n = 5156)	1.28 (1.11–1.48)
Mid-regional proatrial natriuretic peptide (n = 5255)	1.13 (0.98–1.31)
Mid-regional proadrenomedullin (n = 5254)	1.16 (1.00–1.34)

The following variables were entered in the multivariable analysis besides each respective plasma biomarker: Age at study entry, BMI, sex, current smoking, diabetes mellitus, hypertension, and total cholesterol. * Hazard ratios (HRs) were expressed per one SD increment of each respective log-transformed plasma biomarker. LEAD: lower extremity arterial disease, CI: Confidence interval. Table modified from Fatemi et al. [30,31].

**Table 4:** Hazard ratios for plasma biomarkers associated with incident symptomatic and asymptomatic CAS.

Plasma Biomarkers	Symptomatic CAS n = 56. HR * (95% CI)	Asymptomatic CAS n = 54. HR * (95% CI)
Lipoprotein-associated phospholipase A_2_ (mass)	1.28 (0.97–1.69)	0.87 (0.64–1.18)
Lipoprotein-associated phospholipase A_2_ (activity)	1.34 (0.99–1.81)	0.95 (0.68–1.33)
Proneurotensin	0.90 (0.63–1.28)	0.96 (0.70–1.30)
Mid-regional proadrenomedullin	1.40 (1.13–1.73)	0.98 (0.69–1.40)
Mid-regional proatrial natriuretic peptide	1.12 (0.83–1.52)	0.79 (0.58–1.09)
N-terminal pro-B-type natriuretic peptide	1.59 (1.20–2.11)	1.08 (0.80–1.47)
Copeptin	1.35 (0.88–2.06)	1.16 (0.80–1.67)
Cystatin C	1.21 (1.02–1.43)	0.75 (0.50–1.10)
C-reactive protein	1.53 (1.13–2.05)	1.08 (0.80–1.46)

The following variables analyzed at baseline were entered in the multivariable analysis besides each respective plasma biomarker: Age, sex, BMI, current smoking, diabetes mellitus, hypertension, and cholesterol. CAS: carotid artery stenosis. Asymptomatic patients were excluded when assessing symptomatic CAS, and symptomatic patients were excluded when assessing asymptomatic CAS. * Hazard ratios (HRs) were expressed per one SD increment of each respective log-transformed plasma biomarker. CI: Confidence interval. Table modified from Fatemi et al. [33].

**Table 5:** Hazard ratios for plasma biomarkers associated with incident-isolated AD and incident-isolated AAA.

Plasma Biomarker	Incident AD (Free from Incident AAA) HR * (95% CI)	Incident AAA (Free from Incident AD) HR * (95% CI)
Lipoprotein-associated phospholipase A_2_ (activity)	1.12 (1.04–1.19)	1.53 (1.11–2.11)
Lipoprotein-associated phospholipase A_2_ (mass)	1.05 (0.99–1.12)	1.53 (1.14–2.04)
Copeptin	1.09 (1.01–1.17)	0.98 (0.70–1.39)
Mid-regional proadrenomedullin	1.17 (1.10–1.25)	1.47 (1.15–1.88)
Mid-regional proatrial natriuretic peptide	1.03 (0.97–1.11)	1.01 (0.71–1.43)
N-terminal pro-B-type natriuretic peptide	1.16 (1.08–1.24)	1.13 (0.80–1.60)
Cystatin C	1.17 (1.11–1.23)	1.13 (0.82–1.55)
Proneurotensin	1.07 (1.02–1.13)	1.09 (0.85–1.40)
C-reactive protein	1.17 (1.10–1.25)	1.22 (0.88–1.68)

Adjusted for age, sex, BMI, current smoking, hypertension, total cholesterol, and each respective plasma biomarker. AD: atherosclerotic disease, AAA: abdominal aortic aneurysm. * Hazard ratios (HRs) were expressed per one SD increment. Participants with incident AAA were excluded when assessing participants with incident AD, and participants with incident AD were excluded when assessing participants with incident AAA. CI: Confidence interval. Table modified from Acosta et al. [34].

## Data Availability

The raw data supporting the conclusions of this article will be made available by the authors upon request, without undue reservation.
